# Long-Term Treatment with Atypical Antipsychotic Iloperidone Modulates Cytochrome P450 2D (CYP2D) Expression and Activity in the Liver and Brain via Different Mechanisms

**DOI:** 10.3390/cells10123472

**Published:** 2021-12-09

**Authors:** Przemysław J. Danek, Władysława A. Daniel

**Affiliations:** Department of Pharmacokinetics and Drug Metabolism, Maj Institute of Pharmacology, Polish Academy of Sciences, Smętna 12, 31-343 Kraków, Poland; danek@if-pan.krakow.pl

**Keywords:** iloperidone, prolonged administration, brain, liver, cytochrome P450 2D expression/activity

## Abstract

*CYP2D* enzymes engage in the synthesis of endogenous neuroactive substances (dopamine, serotonin) and in the metabolism of neurosteroids. The present work investigates the effect of iloperidone on CYP2D enzyme expression and activity in rat brains and livers. Iloperidone exerted a weak direct inhibitory effect on CYP2D activity in vitro in the liver and brain microsomes (K_i_ = 11.5 μM and K_i_ = 462 μM, respectively). However, a two-week treatment with iloperidone (1 mg/kg ip.) produced a significant decrease in the activity of liver CYP2D, which correlated positively with the reduced CYP2D1, CYP2D2 and *CYP2D4* protein and mRNA levels. Like in the liver, iloperidone reduced CYP2D activity and protein levels in the frontal cortex and cerebellum but enhanced these levels in the nucleus accumbens, striatum and substantia nigra. Chronic iloperidone did not change the brain *CYP2D4* mRNA levels, except in the striatum, where they were significantly increased. In conclusion, by affecting CYP2D activity in the brain, iloperidone may modify its pharmacological effect, via influencing the rate of dopamine and serotonin synthesis or the metabolism of neurosteroids. By elevating the CYP2D expression/activity in the substantia nigra and striatum (i.e., in the dopaminergic nigrostriatal pathway), iloperidone may attenuate extrapyramidal symptoms, while by decreasing the CYP2D activity and metabolism of neurosteroiods in the frontal cortex and cerebellum, iloperidone can have beneficial effects in the treatment of schizophrenia. In the liver, pharmacokinetic interactions involving chronic iloperidone and CYP2D substrates are likely to occur.

## 1. Introduction

Schizophrenia is a serious mental illness consisting of positive (psychotic), negative (anhedonia, emotional withdrawal), and cognitive disorders [1]. To treat this mental illness, a number of antipsychotic drugs are currently available on the market. Iloperidone is an atypical neuroleptic drug approved for the treatment of acute schizophrenia in adult patients. Iloperidone is a piperidinyl-benzisoxazole derivate structurally related to risperidone, paliperidone and ziprasidone [2]. This atypical neuroleptic produces an antagonistic effect through high-affinity binding to serotonin 5-HT_2A_, dopamine D_2_ and D_3_ receptors and adrenergic α_1_ and α_2_ receptors, moderate-affinity binding to dopamine D_4_ and serotonin 5-HT_2C_, 5-HT_6_ and 5-HT_7_, and low-affinity binding to serotonin 5-HT_1A_, dopamine D_1_, and histamine H_1_ receptors, and has no appreciable affinity for muscarinic receptors [3]. Thus, the receptor profile of iloperidone suggests its efficacy in alleviating the positive and negative symptoms of schizophrenia, as well as efficacy in reducing cognitive symptoms, with minimal side effects [4,5,6,7,8]. Iloperidone is extensively metabolized in the liver, by hydroxylation and carbonyl reduction (mediated by CYP2D6), and to a lesser degree *O*-dealkylation (mediated by CYP3A4) [9].

Cytochrome P450 (CYP) enzymes are a superfamily of membrane-bound hemoproteins responsible for the oxidative metabolic processes of xenobiotics (e.g., drugs, environmental pollutants, promutagenes) and endogenous compounds (hormones, bile acid, vitamins, neurotransmitters) [10,11,12].

The CYP-catalyzed metabolism of drugs occurs predominantly in the liver, where CYPs are highly expressed and are largely responsible for systemic drug and metabolite levels, but many CYP enzymes have been detected in a number of extrahepatic tissues, including the brain. In the brain, the expression of CYP enzymes is generally lower than in the liver (0.5–2% of the hepatic level), so its contribution to the overall clearance of xenobiotics is limited, but the regulation of the local metabolism and concentration of endogenous and exogenous compounds and thus the neuroprotective effect of CYP may be of importance [13,14,15]. Hepatic CYPs are generally expressed in the endoplasmic reticulum, while brain CYPs are found also on mitochondrial membranes, plasma membranes and other cell compartment membranes [16]. 

Different CYP2D enzymes have been identified in numerous species including humans, rats and mice. Humans only have one functional CYP2D form (CYP2D6), while rats have six forms (CYP2D1-5 and CYP2D18) with unique substrate specificity, metabolism, expression and inhibitors [17]. CYP2D1 and CYP2D2 are the most abundant forms in rat livers, while CYP2D4 is primarily expressed in rat brains [18,19]. As compared to humans and rats, mice have nine *Cyp2d* genes: *Cyp2d9*, *Cyp2d10*, *Cyp2d11*, *Cyp2d12*, *Cyp2d13*, *Cyp2d22*, *Cyp2d26*, *Cyp2d34* and *Cyp2d40* [20]. The region- and cell-specific expression of brain CYP2D is relatively consistent across species, with marked similarities in regions with the highest brain CYP2D expression (the substantia nigra and cerebellum) [21,22]. 

Liver CYP2D metabolizes numerous xenobiotic compounds, including drugs that target the brain, such as antidepressants (e.g., amitriptyline, fluoxetine), antipsychotics (e.g., haloperidol, thioridazine, iloperidone), analgesics (e.g., codeine, tramadol), β-blockers (e.g., propranolol, metoprolol), neurotoxins (e.g., 1-methylo-4-phenyl-1,2,3,6-tetrahydropyridine (MPTP), and tetrahydroisoquinolines (TIQ)) [12,23]. Moreover, CYP2D catalyzes alternative pathways of dopamine and serotonin synthesis in the brain [24,25,26], and the local metabolism of neurosteroids [27], arachidonic acid [28], and endogenous opioids [29]. 

An in vitro study on human liver microsomes and cDNA-expressed CYP enzymes showed a direct inhibitory effect of iloperidone on the activity of CYP2D6 and CYP3A4 via competitive and noncompetitive mechanisms, respectively [30], and its diminishing effect on the CYP3A4 expression and activity in a hepatocyte culture [31]. However, a possible effect on brain CYP2D expression in vivo, produced by a chronic treatment with iloperidone, has not been studied as yet, although neuroleptic therapies of psychiatric disorders have to be continued for months or years. Therefore, the aim of our present work was to investigate the effects of chronic treatment of iloperidone on the CYP2D enzyme expression and activity in the selected rat brain structures and liver.

## 2. Materials and Methods

### 2.1. Animals

All animal procedures were performed following the European regulations for animal experimentation on the Protection of Animals Used for Scientific Purposes (EU Directive 2010/63/EU). The experimental protocols were approved by the Local Ethics Commission for Experimentation on Animals at the Maj Institute of Pharmacology, Polish Academy of Sciences, Kraków. Animals were maintained on a 12 h light/dark cycle (light on at 08.00 h) at 22 ± 2 °C and 50 ± 5% humidity with free access to typical laboratory food and tape water. Male Wistar Han rats (age: 3 months; bodyweight: 270–300 g) were purchased from Charles River Laboratories (Sulzfeld, Germany). 

### 2.2. Drugs and Chemicals

Reagents for determining the activity of CYP2D enzymes in brain and liver microsomes (glucose-6-phosphate, glucose-6-phosphate-dehydrogenase, NADP) and specific CYP2D substrate and its metabolite (bufuralol and 1′-hydroxybufuralol) came from Sigma (St. Louis, MO, USA). The SignalBoost^TM^Immunoreaction Enhancer Kit used for the dilution of primary and secondary antibodies was supplied by Millipore (Burlington, MA, USA). Laemmli sample buffer used for the dilution of samples was obtained from Bio-Rad (Hercules, CA, USA). RNA was isolated with a Total RNA Mini kit from A&A Biotechnology (Gdynia, Poland). Life Technologies (Carlsbad, CA, USA) provided a High-Capacity cDNA Reverse Transcription Kit, TaqMan assay and the TaqMan Gene Expression Master Mix. Merck (Darmstadt, Germany) provided the organic solvents with HPLC purity.

### 2.3. Animal Treatment and Preparation of Brain and Liver Microsomes

To differentiate between the direct effect of iloperidone on the activity of CYP2D and changes evoked by their chronic in vivo treatment, two experimental approaches were applied. To study the direct effect on the CYP2D protein (inhibition), iloperidone was added in vitro to control brain or liver microsomes (Experiment I). To study the possible influence of iloperidone on CYP2D expression, the drug was administered to rats in vivo for two weeks (Experiment II). 

Rats (*n* = 12) were injected intraperitoneally once a day with a pharmacological dose of iloperidone (1 mg/kg ip.) or vehicle (1% Tween 80 in sterile water) for a period of two weeks. The dose administered was consistent with previous pharmacological studies on rats [32,33,34], and the dose was active in neurochemical and behavioral paradigms. Rats were killed by decapitation 24 h after the last dose. Brains and livers were removed and the selected brain structures (in accordance with the Paxinos and Watson atlas [35]), receiving dopaminergic and/or serotonergic innervation (the nucleus accumbens, frontal cortex, substantia nigra, striatum, hippocampus, hypothalamus, brain stem, cerebellum, and the remainder of the brain), were isolated and frozen in dry ice and stored at −80 °C until use. Microsomal fraction from the whole control brains, selected brain structures or livers was prepared by differential centrifugation, according to Hiroi et al. [36] and Haduch et al. [37]. Brain microsomes were immediately used to determine CYP2D activity, while those of liver microsomes were stored at −80 °C until use.

### 2.4. CYP2D Enzyme Activity in Brain and Liver Microsomes

The CYP2D activity was determined using the CYP2D specific reaction, i.e., 1′-hydroxylation of bufuralol in microsomes prepared from the brains or livers of control rats (Experiment I) and iloperidone-treated animals (Experiment II), as described previously [38,39]. The metabolism of bufuralol was investigated in terms of the linear dependence of product formation on time, substrate and protein concentration.

In Experiment I (inhibition studies), the experiments were performed on the brain microsomes from the whole brain (2 mg of protein/mL) or liver microsomes (0.5 mg of protein/mL) obtained from control rats. The specific reaction, i.e., 1′-hydroxylation of bufuralol, proceeded at the substrate concentrations of 50, 100 and 200 µM for brain microsomes or 5, 10 and 20 µM for liver microsomes, in the absence or presence of in vitro added iloperidone (1–250 µM for liver microsomes or 25–500 µM for brain microsomes), and was studied under the in vitro conditions described below. 

In the study with iloperidone-treated animals (Experiment II), the bufuralol 1′-hydroxylation reaction proceeded in a system containing brain microsomes derived from selected brain structures of 1–3 rats (ca. 0.4 mg of protein/mL for the nucleus accumbens, 0.7 mg of protein/mL for the hippocampus and the substantia nigra, 1.2 mg of protein/mL for the hypothalamus, 1.5 mg of protein/mL for the striatum, the brain stem and the cerebellum and 2 mg of protein/mL for the frontal cortex and remainder of the brain) or liver microsomes (0.5 mg of protein/mL), potassium phosphate buffer (2 mM, pH = 7.4), NADP (1.6 mM), MgCl_2_ (4 mM), glucose 6-phosphate (5 mM) and glucose 6-phosphate-dehydrogenase (2.5 U in every sample), as described earlier [37,38]. Bufuralol was added to the incubation medium containing brain microsomes at a concentration of 125 µM or liver microsomes at a concentration of 10 µM to the final volume of 0.4 mL. The total level of microsomal protein was measured by the method of Lowry et al. [40] using bovine serum albumin as a standard. 

In all experiments, the amount of 1′-hydroxybufuralol formed from bufuralol was measured by an HPLC method with fluorometric detection [41].

### 2.5. Evaluation of CYP2D Protein in Brain and Liver Microsomes 

The CYP2D protein levels in microsomes from the brains and livers of control and iloperidone-treated animals were quantified by Western blotting, as previously described [42,43]. Briefly, microsomal proteins (10 μg of brain and liver microsomes per each sample) were separated using an SDS polyacrylamide gel electrophoresis, and then the protein bands were transferred onto nitrocellulose membranes (Amersham Protran, Merck KGaA, Darmstadt, Germany). The polyclonal rabbit anti-rat CYP2D4 antibody (donated by Dr. Y. Funae from the University of Osaka, Medical School, Japan) and polyclonal rabbit anti-human CYP2D6 antibody (Fine Test, Wuhan, China) were used as the primary antibodies for CYP2D4 in brain microsomes and CYP2D enzymes in liver microsomes, respectively. Horseradish peroxidase-labeled goat anti-rabbit IgG was used as a secondary antibody (Vector Laboratories, Burlingame, CA, USA). For the estimation of β-actin level, the primary mouse polyclonal anti-rat β-actin antibody (Sigma, St. Louis, MO, USA) and goat anti-mouse antibody (Jackson ImmunoResearch, West Grove, PA, USA) were used. Rat cDNA-expressed CYP2D4 (2.5 µg, Cypex, Dundee, Scotland, UK) and human CYP2D6 (1 µg, Gentest Corp. Woburn, MA, USA) were used as standards. The band intensity of the CYP2D protein was evaluated with the Luminescent Image Analyzer LAS-1000 and Image Gauge 3.11 programs (Fuji Film, Tokyo, Japan). The collected data were normalized to protein loading based on the β-actin levels.

### 2.6. Examination of the Expression of Genes Coding for CYP2D Enzymes in the Brain and Liver

A total RNA mini kit was used to extract RNA from liver tissues or 10,000 g tissue pellets derived from selected brain structures. RNA (1 μg) was used to prepare cDNA with a High-Capacity cDNA Reverse Transcription Kit. The obtained cDNA was used as a template for quantitative real-time PCR with a TaqMan Gene Expression Assay using TaqMan Gene Expression Master Mix, TaqMan type probes and primers (*CYP2D1* (Rn01775090_mH), *CYP2D2* (Rn00562419_m1), *CYP2D4* (Rn00593393_m1), and gene encoding β-actin *ACTB* (Rn00667869_m1)), and a Bio-Rad CFX96 PCR system (Bio-Rad, Hercules, CA, USA). Gene expression was determined using the 2-delta Ct method and *ACTB* expression as a reference, as reported previously [44].

### 2.7. Data Analysis

Dixon plots (1/V vs. I) indicating K_i_ values show the in vitro direct inhibitory effects of iloperidone on the CYP2D enzyme activity in brain/liver microsomes. The Lineweaver–Burk plots (1/V vs. 1/S) show the mechanism of CYP2D enzyme inhibition by iloperidone. A rise in the K_m_ value at no change in the V_max_ value testifies to competitive inhibition (Program Sigma Plot 12.3 Enzyme Kinetics, Systat Software Inc., San Jose, CA, USA). 

The results of chronic neuroleptic treatment are reported as the mean ± S.E.M. The statistical significance of alterations in enzyme activity, protein level or gene expression was calculated using a Student’s *t*-test compared to control value (GraphPad Prism 8.0; GraphPad Software Inc., San Diego, CA, USA). The changes found in the enzyme activity, protein level or mRNA were considered as statically significant when *p* < 0.05. 

## 3. Results

### 3.1. Inhibition of CYP2D Activity by Iloperidone in Control Brain and Liver Microsomes (Experiment I)

**Figure 1:**
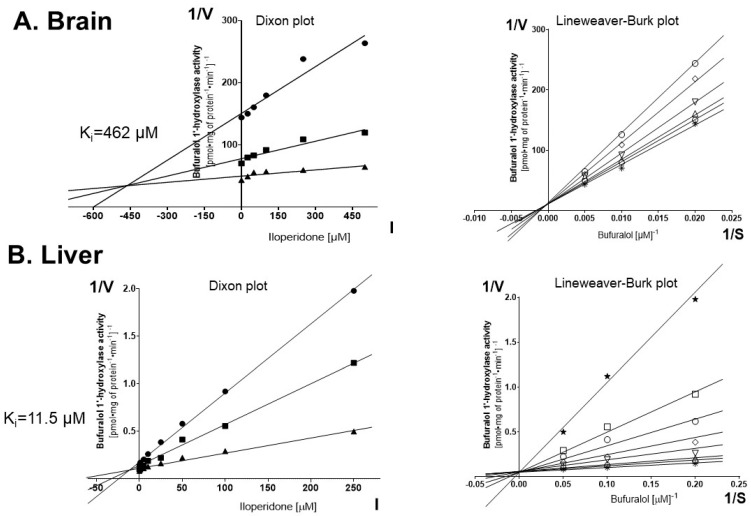
The effects of iloperidone added in vitro to pooled brain or liver microsomes (*n* = 10) on the activity of CYP2D determined as the rate of bufuralol 1′-hydroxylation. Each point shows the mean value of two independent analyses. (**A**) Brain. Kinetic parameters: K_m_ = 492.1 μM, V_max_ = 0.076 pmol∙mg protein^−1^∙min^−1^, K_i_ = 462 µM. Dixon plot: bufuralol concentrations of 50 µM (●), 100 µM (■) and 200 µM (▲). Lineweaver-Burk plot: control—no asenapine (✱); the asenapine concentrations of 25 µM (⎔), 50 µM (△), 100 µM (▽), 250 µM (◊) and 500 µM (○). (**B**) Liver. Kinetic parameters: K_m_ = 8.26 μM, V_max_ = 15.45 pmol∙mg protein^−1^∙min^−1^, K_i_ = 11.5 µM. Dixon plot: bufuralol concentrations of 5 µM (●), 10 µM (■) and 20 µM (▲). Lineweaver–Burk plot: control—no asenapine (✱); the asenapine concentrations of 1 µM (⎔), 5 µM (△), 10 µM (▽), 25 µM (◊), 50 µM (○), 100 µM (◻) and 250 µM (★). V, velocity of the reaction; I, the inhibitor (iloperidone) concentration; S, the substrate (bufuralol) concentration.

To investigate a possible direct effect of iloperidone on the activity of CYP2D enzymes, the specific reaction (bufuralol 1′-hydroxylation) assays were conducted in vitro with varied concentrations of the neuroleptic (Figure 1). The Dixon plots of bufuralol 1′-hydroxylation in the absence or presence of the tested drug indicated that iloperidone exerted a very weak inhibitory effect on the CYP2D activity in rat brain microsomes (K_i_ = 462 μM) (Figure 1A) and a more pronounced effect on the CYP2D activity in the liver microsomes (K_i_ = 11.5 μM) (Figure 1B). The Lineweaver–Burk plots revealed that in both brain and liver microsomes the neuroleptic inhibited the enzyme activity via a competitive mechanism (Figure 1A,B).

### 3.2. The Effect of Chronic Iloperidone Treatment on the CYP2D Activity in the Brain and Liver Microsomes (Experiment II) 

**Figure 2:**
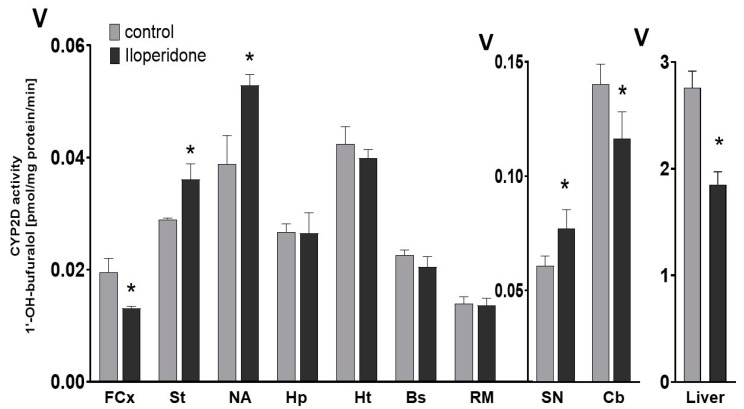
The influence of the two-week treatment with iloperidone on the CYP2D activity measured in microsomes derived from the selected brain structures or liver. The presented values are the means ± S.E.M. of 12 samples (from 12 animals) for the cerebellum, remainder of brain and the liver, of 6 samples (each sample contained 2 pooled brain structures from 2 animals) for the frontal cortex, brain stem, striatum and hippocampus, and of 4 samples (each sample contained 3 pooled brain structures from 3 animals) for the hypothalamus, nucleus accumbens and substantia nigra. Student’s *t*-test: * *p* < 0.05 vs. control group. FCx—the frontal cortex, St—the striatum, NA—the nucleus accumbens, Hp—the hippocampus, Ht—the hypothalamus, Bs—the brain stem, SN—the substantia nigra, Cb—the cerebellum and RM—the remainder.

The obtained results indicate that the chronic administration of iloperidone exerts a significant broad effect on the activity of CYP2D in different regions of the brain (Figure 2, Table 1). A two-week treatment with iloperidone decreased the CYP2D activity in the frontal cortex (down to 67% of the control) and cerebellum (down to 79% of the control). On the other hand, the prolonged administration of iloperidone enhanced the enzyme activity in the striatum (up to 124% of the control), the nucleus accumbens (up to 136% of the control) and in the substantia nigra (up to 126% of the control). The tested neuroleptic did not significantly influence the CYP2D activity in the brain stem, hypothalamus, hippocampus and the remainder of the brain. In the liver, the repeated administration of iloperidone reduced the CYP2D activity to 67% of the control (Figure 2). 

### 3.3. The Effect of Chronic Iloperidone Treatment on the CYP2D Protein Level in Microsomes Derived from the Brain and Liver (Experiment II)

**Figure 3:**
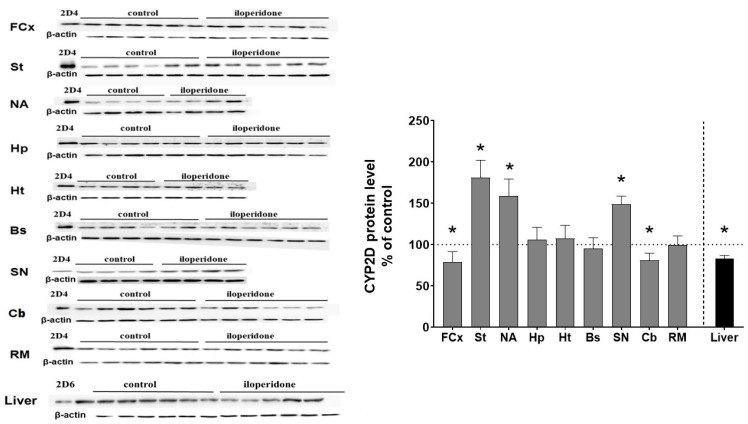
The effect of two-week treatment with iloperidone on the CYP2D protein levels measured in microsomes derived from the selected brain structures or liver. The presented values are the means ± S.E.M. of 12 samples (from 12 animals) for the cerebellum, remainder of brain and the liver, of 6 samples (each sample contained 2 pooled brain structures from 2 animals) for the frontal cortex, brain stem, striatum and hippocampus or of 4 samples (each sample contained 3 pooled brain structured from 3 animals) for the hypothalamus, nucleus accumbens and substantia nigra. The representative CYP2D protein bands of the Western blot analysis are shown. Brain or liver microsomal protein (10 µg) was subjected to Western blot analysis. cDNA expressed CYP2D4 protein (Bactosomes) and cDNA expressed CYP2D6 protein (Supersomes) was used as a positive control. Student’s *t*-test: * *p* < 0.05 vs. control group. FCx—the frontal cortex, St—the striatum, NA—the nucleus accumbens, Hp—the hippocampus, Ht—the hypothalamus, Bs—the brain stem, SN—the substantia nigra, Cb—the cerebellum and RM—the remainder.

The CYP2D protein level was measured by Western blot analysis in the microsomal fraction from different brain regions of control and iloperidone-treated rats. In the rat, CYP2D1 and CYP2D2 are the most abundant CYP forms in the liver, while CYP2D4 is primarily expressed in the brain [18,19]. As shown in Figure 3, iloperidone exerted a significant effect on the CYP2D protein level in the brain. The observed changes in CYP protein levels correlated positively with those in the enzyme activity after chronic treatment with the tested drug (Table 1). Iloperidone reduced the CYP2D protein level in the frontal cortex (down to 78% of the control) and the cerebellum (down to 80% of the control), but enhanced the enzyme protein level in the striatum (up to 181% of the control), the nucleus accumbens (up to 159% of the control) and the substantia nigra (up to 148% of the control). The tested neuroleptic did not affect the CYP2D4 protein level in the brain stem, hypothalamus, hippocampus and the remainder of the brain.

On the other hand, iloperidone significantly reduced the CYP2D protein level in the liver after chronic administration, down to 86% of the control (Figure 3). The diminished CYP2D protein level positively correlated with the reduced CYP2D enzyme activity after chronic neuroleptic treatment.

### 3.4. The Effect of Chronic Iloperidone Treatment on the CYP2D Gene Expression in the Brain and Liver (Experiment II)

**Figure 4:**
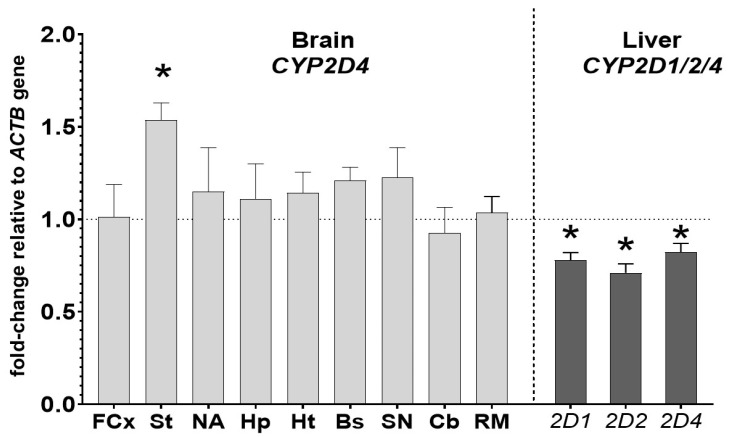
The influence of two-week treatment with iloperidone on the mRNA levels of the *CYP2D4* gene in the selected brain structures and on the mRNA levels of the *CYP2D1*, *CYP2D2* and *CYP2D4* genes in the liver. The results are expressed as the fold change compared to the *ACTB* housekeeping gene. The presented values are the mean fold change quantified by the comparative delta-delta Ct method for the control and iloperidone-treated rats (mean ± S.E.M. of 12 samples (from 12 animals) for the cerebellum, remainder of brain and the liver, of 6 samples (each sample contained 2 pooled brain structures from 2 animals) for the frontal cortex, brain stem, striatum and hippocampus or of 4 samples (each sample contained 3 pooled brain structured from 3 animals) for the hypothalamus, nucleus accumbens and substantia nigra). Student’s *t*-test: * *p* < 0.05 vs. control group. FCx—the frontal cortex, St—the striatum, NA—the nucleus accumbens, Hp—the hippocampus, Ht—the hypothalamus, Bs—the brain stem, SN—the substantia nigra, Cb—the cerebellum and RM—the remainder.

The mRNA levels of *CYP2D* genes were measured in the brain structures (*CYP2D4* mRNA) and liver (*CYP2D1*, *CYP2D2* and *CYP2D4* mRNAs) to further investigate the molecular mechanism of the observed changes in the CYP activity and protein level. The mRNA levels of the main liver CYP2D enzymes, i.e., *CYP2D1* and *CYP2D2* mRNAs, and of the main brain CYP2D enzyme, i.e., *CYP2D4* mRNA, were measured. In addition, *CYP2D4* mRNA was also measured in the liver to compare the regulation of *CYP2D4* genes by iloperidone in the two studied organs. In the brain, *CYP2D4* mRNA was increased only in the striatum (up to 153% of the control) after the two-week treatment with the iloperidone. In the other examined brain structures, the *CYP2D4* mRNAs were not significantly changed (Figure 4), though some tendency to increase (e.g., in the substantia nigra) was observed. In the liver, the investigated neuroleptic produced a significant decrease in the mRNA levels of the *CYP2D1*, *CYP2D2* and *CYP2D4* genes, down to 78%, 71% and 82%, respectively (Figure 4, Table 1).

## 4. Discussion

This is the first report showing parallel changes in the CYP2D activity and expression in different brain regions and in the liver under prolonged iloperidone treatment in rats. The obtained results provide evidence that a two-week iloperidone treatment produces different effects on the brain and on the liver CYP2D, and the effect on brain CYP2D depends on the cerebral structure (Table 1).

As mentioned in the introduction, in vitro studies on human liver microsomes have indicated that iloperidone has the potential to inhibit the CYP2D6 activity through direct interaction with the enzyme protein via competitive mechanism with the Ki value of 2.9 μM, which may be of pharmacological importance [30,45]. In the present study, using rat liver microsomes, the inhibitory effect of iloperidone was weaker, as indicated by the Ki value of 11.5 μM. Since the pharmacological plasma concentration of iloperidone in rats after the applied dose is low (Cmax = 0.8 μM) [46], the neuroleptic does not seem to reach the concentration close to its Ki value in the liver to become of pharmacological significance in the rat in vivo, in spite of its lipophilicity and a weakly basic character (logP = 4.43, pKa = 7.91).

The potential of iloperidone to inhibit CYP2D in the rat brain is even weaker than in the liver (Ki = 462 μM), which indicates no direct inhibitory effect of iloperidone on the CYP2D activity in the brain in vivo, compared to a possible weak effect in the liver. These differences may result from the diverse composition of CYP2D enzymes in the brain and liver, as well as a higher degree of nonspecific binding due to a higher amount of phospholipids in the brain (discussed in [37,47]). It has been shown in an in vitro study on brain slices that the tissue/medium concentration ratio for iloperidone was 17 [48]. Thus, the concentrations of iloperidone in the brain will be much higher than in blood plasma [49], but still lower by far than that of the respective Ki (462 μM) to become of pharmacological significance in vivo.

However, iloperidone can affect the CYP2D activity in the liver and brain as a result of its chronic administration in vivo. The observed decrease in the activity of liver CYP2D after the two-week iloperidone treatment corresponds well with the reduced CYP2D1, CYP2D2 and CYP2D4 protein levels in the rat liver microsomes and *CYP2D1*, *CYP2D2* and *CYP2D4* mRNA levels, which indicates that the negative regulation of activity/expression of liver CYP2D enzymes by iloperidone proceeds at the transcriptional level. Although hepatic CYP2D levels are considered to be primarily influenced by genetic factors, recent studies indicate that their regulation at a transcriptional level is also possible by continuous treatment with some drugs, such as atypical neuroleptics, which has also been observed for asenapine [42].

Chronic treatment with iloperidone also affects brain CYP2D in a region-dependent manner. Like in the liver, iloperidone reduces the CYP2D activity and protein levels in the frontal cortex and cerebellum, but in contrast, the neuroleptic enhances those levels in the striatum, nucleus accumbens and substantia nigra. The observed regional changes in the CYP2D activity positively correlate with those in the CYP2D4 protein level; however, in most cases the enzyme was regulated at the post-transcriptional level, since a two-week treatment with iloperidone did not change the *CYP2D4* mRNA level. The only exception was the striatum, where the level of *CYP2D4* mRNA was significantly increased, as was the enzyme protein and activity levels. Interestingly, like other drugs/xenobiotics (clozapine, asenapine, nicotine, ethanol and toluene), iloperidone may induce brain CYP2D at a post-transcriptional level, though with a different regional pattern of induction [23,37,47,50,51,52,53].

It is well known that CYP2D is differently regulated in the liver and brain by xenobiotics, possibly due to the various levels of transcription factors, receptor signal transduction pathways and blood–brain permeability for endo- and exogenous compounds [13,54]. This may also be a reason for the different effects of iloperidone and the previously investigated atypical neuroleptic asenapine on brain regional CYP2D expression and activity, with the same effect on liver CYP2D [47]. Both neuroleptics potently antagonize dopaminergic D_2_ and D_3_, adrenergic α_1_ and α_2_ and serotonergic 5-HT_2A_ receptors, but asenapine also binds with a high affinity to other serotonergic receptors (5-HT_1A_, 5-HT_2B_, 5-HT_2C_, 5-HT_6_ and 5-HT_7_) and histaminergic H_1_ receptors [3,7,55,56]. These differences in the receptor profiles and neuronal transduction signals produced by the two neuroleptics may be important for the regulation and functioning of the CYP2D enzymes in particular brain regions (Table 2).

The investigated brain structures are not homogenous. For example, the nucleus accumbens core and shell are innervated similarly, but there are some differences in their morphology and function. The different neurochemistry and signals received by GABAergic medium spiny projecting neurons in the shell and core during neuroleptic treatment might affect the expression/activity of CYP2D in the two substructures of the nucleus accumbens neurons [57,58,59,60]. It was shown that atypical neuroleptics increased dopamine concentrations to a greater extent in the shell than in the core [61]. Furthermore, differences in Fos-like immunoreactivity indicate that the shell of the accumbens may be a site of antipsychotic action [62,63]. Therefore, differences in the activity of CYP2D between these two subregions of the rat nucleus accumbens are conceivable, which will be the subject of further, detailed studies.

Since the distribution of CYP enzymes in the brain is heterogeneous, it is possible that different drug concentrations in particular brain regions influence the local enzyme activity to a different extent [16]. Therefore, regional changes in brain CYP2D activity may contribute to differences in individual responses to centrally acting CYP2D substrates that are not reflected in peripheral drug and metabolite levels [64]. There is little research on the regulation of CYP2D enzymes in the brain, and the effect of chronic iloperidone treatment on the activity/expression of CYP2D enzymes in selected brain structures has not been studied thus far. However, it seems that various levels of transcription factors, receptor signal transduction pathways and blood–brain permeability for a drug and its metabolites in particular brain structures may influence the expression of CYP2D at particular stages of biosynthesis and function (activity) [13,54].

The observed effects of iloperidone on brain CYP2D might be partly due to a reduced level of growth hormone (GH) after iloperidone treatment. In our recent work, we have reported that two-week treatment with iloperidone reduces serum GH level in rats [43]. GH induces the tissue-specific alteration of PPARs, which are highly expressed in the brain in a region-selective manner. Zhang et al. [65] reported that pulsatile GH treatment decreased the *PPARα* mRNA level and increased the mRNA levels of *CYP2D6* and *PPARγ* in SH-SY5Y human neuroblastoma cells. CYP2D enzymes were positively regulated by PPARγ but negatively by PPARα. It is feasible that by reducing the GH level, iloperidone could selectively downregulate brain CYP2D by influencing PPARs in different brain regions. However, pulsatile pituitary GH, which is released into the blood, does not easily penetrate the blood–brain barrier [66,67], so it seems that the above-described effect of GH on neuronal CYP2D in vitro would rather depend on locally synthesized GH in the brain in vivo.

Moreover, the brain CYP2D enzymes contribute to the de novo synthesis of endogenous morphine from various sequentially biotransformed precursors including tyramine, dopamine, 3,4-dihydroxy-L-phenylalanine and reticuline [29,68,69]. At higher (nanomolar) concentrations, observed under traumatized conditions, endogenous morphine stimulates the μ_3_ opiate receptor and, through the CA^2+^-stimulated activation of cNOS, is responsible for releasing NO [70], which can downregulate gene expression or bind to enzyme proteins [71]. However, it seems unlikely that, by moderately increasing the CYP2D activity in the brain (in the substantia nigra, striatum and nucleus accumbens), iloperidone could accelerate the production of morphine to such a degree as to enhance its levels from picomolar to nanomolar, required for the activation of μ_3_ receptors in the brain [72,73].

The observed changes in the expression of CYP2D enzymes and activity after prolonged iloperidone treatment may be of pharmacological importance in the treatment of schizophrenia. By increasing CYP2D activity, iloperidone may accelerate the local synthesis of dopamine via tyramine hydroxylation or of serotonin via 5-methoxytryptamine O-demethylation, i.e., alternative pathways of neurotransmitter synthesis [24,25]. It was found that CYP2D in the striatum was colocalized with the dopamine transporter on membranes of dopaminergic neurons [74], which implies that CYP2D is implicated in the synthesis and release of dopamine. Since iloperidone elevated the CYP2D expression and activity in both the substantia nigra and striatum, i.e., in the dopaminergic nigrostriatal pathway, it is possible that this biochemical effect may result in the attenuation of neuroleptic-induced extrapyramidal symptoms. On the other hand, by reducing the CYP2D activity in other brain regions (the frontal cortex, cerebellum), iloperidone may slow down the oxidative metabolism of neurosteroids by inhibiting their 21-hydroxylation metabolic pathway [27,75], and thus may exert beneficial effects on the symptoms of schizophrenia by affecting neuroplasticity and improving memory and cognitive functions [76,77].

## 5. Conclusions

In conclusion, iloperidone exerts a wide range of actions on cytochrome CYP2D, which may be of pharmacological importance in the treatment of schizophrenia. Iloperidone can regulate the expression and activity of CYP2D through various molecular mechanisms, including the weak direct inhibitory effect it has on the CYP2D protein/activity in the liver, as well as transcriptional or post-transcriptional mechanisms in the liver and brain. The above-mentioned CYP2D-related biochemical effects of iloperidone in the brain may have a beneficial influence on the therapeutic and side-effect profile of this drug in the treatment of schizophrenia. In the liver, pharmacokinetic interactions involving iloperidone and CYP2D substrates (e.g., tricyclic antidepressants, codeine, dextromethorphan, neuroleptics, β-blockers) are likely to occur in patients during the co-administration of the above-mentioned drugs.

## Figures and Tables

**Table 1:** The CYP2D activity, protein and mRNA levels in the selected brain structures and liver.

Tissue		CYP2D Activity (% of Control)	CYP2D Protein Level (% of Control)	*CYP2D* mRNA Level (% of Control)		
				*CYP2D1*	*CYP2D2*	*CYP2D4*
Brain structures	FCx	**67 ± 2.7** ↓ *	**78 ± 12.4** ↓ *	n.t.	n.t.	101 ± 17.5
	St	**124 ± 9.5** ↑ *	**181 ± 21.1** ↑ *	n.t.	n.t.	**153 ± 9.4** ↑ *
	NA	**136 ± 5.1** ↑ *	**159 ± 20.3** ↑ *	n.t.	n.t.	115 ± 23.8
	Hp	99 ± 13.6	105 ± 14.9	n.t.	n.t.	111 ± 18.9
	Ht	93 ± 4.7	107 ± 15.9	n.t.	n.t.	114 ± 11.2
	Bs	90 ± 8.1	95 ± 12.8	n.t.	n.t.	121 ± 7.1
	SN	**126 ± 13.7** ↑ *	**148 ± 9.9** ↑ *	n.t.	n.t.	123 ± 16
	Cb	**79 ± 8** ↓ *	**80 ± 8.5** ↓ *	n.t.	n.t.	93 ± 13.8
	RM	97 ± 8.7	99 ± 10.8	n.t.	n.t.	103 ± 8.6
Liver		**67 ± 5.3** ↓ *	**82 ± 4.1** ↓ *	**78 ± 4.1** ↓ *	**71 ± 4.8** ↓ *	**82 ± 4.7** ↓ *

↑, ↓ Increase or decrease, respectively; n.t.—not tested. Student’s *t*-test: * *p* < 0.05 vs. control group. FCx—the frontal cortex, St—the striatum, NA—the nucleus accumbens, Hp—the hippocampus, Ht—the hypothalamus, Bs—the brain stem, SN—the substantia nigra, Cb—the cerebellum and RM—the remainder. For further explanation, see Figure 2, Figure 3 and Figure 4.

**Table 2:** Summary of the effects of chronic treatment with iloperidone and asenapine on the CYP2D protein/activity in the liver and brain.

Drug	Liver	Brain Structures								
		FCx	St	NA	Hp	Ht	Bs	SN	Cb	RM
Iloperidone (D_2_, D_3_, 5-HT_2A_, α_1_, α_2_)	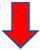	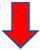	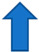	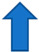	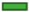	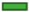	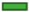	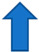	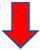	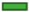
Asenapine (D_1_,D_2_, D_3_, D_4_, 5-HT_1A_, 5-HT_1B_, 5-HT_2A_, 5-HT_2B_, 5-HT_2C_, 5-HT_5A_, 5-HT_6_, 5-HT_7_, α_1_, α_2,_ H_1_)	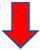	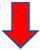	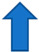	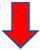	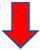	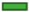	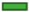	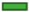	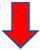	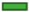

↑, ↓ Increase or decrease, respectively; —no change in the CYP2D activity; receptors mentioned in the brackets display K_i_ below 20 nM [3]; the effect of asenapine on CYP2D is based on Danek et al., 2021 [47]; FCx—the frontal cortex, St—the striatum, NA—the nucleus accumbens, Hp—the hippocampus, Ht—the hypothalamus, Bs—the brain stem, SN—the substantia nigra, Cb—the cerebellum and RM—the remainder.

## Data Availability

The data are contained within the article.
