# Correction: Danek, P.J.; Daniel, W.A. Long-Term Treatment with Atypical Antipsychotic Iloperidone Modulates Cytochrome P450 2D (CYP2D) Expression and Activity in the Liver and Brain via Different Mechanisms. *Cells* 2021, *10*, 3472

**DOI:** 10.3390/cells13242133

**Published:** 2024-12-23

**Authors:** Przemysław J. Danek, Władysława A. Daniel

**Affiliations:** Department of Pharmacokinetics and Drug Metabolism, Maj Institute of Pharmacology, Polish Academy of Sciences, Smętna 12, 31-343 Kraków, Poland; danek@if-pan.krakow.pl

**Text Correction**

There was an error in the original publication [1]. The procedure concerning the liver and brain tissues was not described precisely.

A correction has been made to Section 2.6. Examination of the Expression of Genes Coding for CYP2D Enzymes in the Brain and Liver. The first phrase of the paragraph describing this method has been corrected as below:

A total RNA mini kit was used to extract RNA from liver tissues or 10,000 g tissue pellets derived from selected brain structures.

The authors state that the scientific conclusions are unaffected. This correction was approved by the Academic Editor. The original publication has also been updated.
